# Optimizing Green-Gray Infrastructure for Non-Point Source Pollution Control under Future Uncertainties

**DOI:** 10.3390/ijerph18147586

**Published:** 2021-07-16

**Authors:** Xinyu Dong, Peng Yuan, Yonghui Song, Wenxuan Yi

**Affiliations:** 1 Chinese Research Academy of Environmental Sciences, Beijing 100012, China; dongxinyu@cugb.edu.cn; 2 School of Water Resources and Environment, China University of Geosciences, Beijing 100083, China; yiwenxuan@cugb.edu.cn

**Keywords:** multi-objective optimization, green-gray infrastructure, climate change, urbanization, Non-Point Source Pollution (NPS)

## Abstract

Non-Point Source Pollution (NPS) caused by polluted and untreated stormwater runoff discharging into water bodies has become a serious threat to the ecological environment. Green infrastructure and gray infrastructure are considered to be the main stormwater management measures, and the issue of their cost-effectiveness is a widespread concern for decision makers. Multi-objective optimization is one of the most reliable and commonly used approaches in solving cost-effectiveness issues. However, many studies optimized green and gray infrastructure under an invariant condition, and the additional benefits of green infrastructure were neglected. In this study, a simulation-optimization framework was developed by integrated Stormwater Management Model (SWMM) and Non-dominated Sorting Genetic Algorithm (NSGA-II) to optimize green and gray infrastructure for NPS control under future scenarios, and a realistic area of Sponge City in Nanchang, China, was used as a typical case. Different levels of additional benefits of green infrastructure were estimated in the optimizing process. The results demonstrated that green-gray infrastructure can produce a co-benefit if the green infrastructure have appropriate Value of Additional Benefits (VAB), otherwise, gray infrastructure will be a more cost-effectiveness measure. Moreover, gray infrastructure is more sensitive than green infrastructure and green-gray infrastructure under future scenarios. The findings of the study could help decision makers to develop suitable planning for NPS control based on investment cost and water quality objectives.

## 1. Introduction

Rapid urbanization has resulted in increased impervious surface areas and increased surface pollutant load caused by increasing human production activities [1,2]. Rainfall washes off the surface which contains large pollutant load, resulting in large amounts of pollutants being carried in runoff. The untreated runoff that is discharged directly into receiving water bodies will cause serious Non-Point Source Pollution (NPS) [3]. The extreme rainfall resulted from climate change can further aggravate the challenges faced by urban drainage systems [4]. Climate change and rapid urbanization are the two most influential uncertainties in urban water system management [2,5,6].

Green infrastructure and gray infrastructure are considered as the main measures to control NPS [7,8]. Traditional gray infrastructure, such as storage tanks, can intercept polluted runoff into receiving water bodies at the terminal, which can effectively alleviate the runoff pollution through centralized measures [9,10]. For green infrastructure, when the runoff flows over its surface; meanwhile during realizing the runoff volume control, it can absorb, exfiltrate and deposit contaminants from runoff, so it can further treat polluted runoff [11]. Jia et al. found that the mixed green infrastructures were effective in removing pollutants from runoff, especially in removing NH3-N (ammonia nitrogen) (73%), Total Nitrogen (TN) (74%), Total Phosphorus (TP) (95%) and Total Suspended Solids (TSS) (35%) [12]. Maniquiz-Redillas and Kim evaluated six green infrastructure systems for the removal of heavy metals (Zn, Pb, Cu, Ni, Cr, Cd, and Fe) from polluted runoff and found that all green infrastructure systems were effective in removing particulate-bound metals (Fe, ZN, Pb) while dissolved metals (Cr, Ni, Cu, Cd) were more difficult to remove with green infrastructure systems [13]. Wilson et al. compared the water quality performance of the green infrastructure site and the conventional site from a whole perspective and found green infrastructure could significantly improve the water quality performance of the areas [14].

The implementation of green infrastructure has been regarded as a more sustainable stormwater management, such as Sustainable Urban Drainage Systems (SUDs), Sponge City, and Low Impact Development practices (LID), which can significantly control peak flow, runoff volume, and runoff pollution load at the source through decentralized measures [8,15,16,17]. Although having additional aesthetic and ecological benefits compared to gray infrastructure, many studies have also proven that green infrastructure is more ineffective in controlling high-intensity rainfall of short duration, which makes it become an uneconomic measure correspondingly [18,19,20,21]. Since green and gray infrastructure have their own advantages, more and more studies have begun to focus on studying the combined green-gray infrastructure from a compound perspective [22,23,24,25].

The primary and widespread concern in urban drainage systems planning is how to realize the most cost-effectiveness, and Multi-Objective Optimization (MOO) can be used to obtain Pareto solution sets, which can be more intuitive and easier for decision makers to choose optimal planning scheme [26,27,28]. Therefore, a few studies have proposed frameworks that coupled MOO algorithms with hydrological models for cost-effectiveness optimization of drainage systems. Men et al. proposed a mathematical optimization method for LID practices layout and Sponge City planning by using coupled Preference-Inspired Co-Evolutionary Algorithm using goal vectors (PICEA-g) and SWMM [29]. Eckart et al. developed an integrated framework by coupling Borg Multi-objective Evolutionary Algorithm (Borg MOEA) with SWMM to simulate the optimal LID strategies for stormwater control [30]. Qiu et al. used NSGA-II built into The System for Urban Stormwater Treatment and Analysis Integration (SUSTAIN) model to optimize and evaluate various combined LID measures for NPS control [31]. However, most of the previous studies only considered optimizing green infrastructure rather than gray infrastructure or green-gray infrastructure, and a few other studies considered optimizing gray infrastructure, but only took pipe networks as the goal, and pay more attention to water quantity rather than water quality. Optimizing pipe networks has little practical value, for retrofitting pipe networks is extremely hard to realize in the built areas [32]. In addition, green infrastructure can provide significant indirect additional benefits while meeting direct goals [26], however, the results of many studies that integrated green and gray infrastructure to optimize was incomplete because these additional benefits were neglected [25,27,33]. Moreover, the optimization method of most studies were based on the unchanged condition and merely considered initial construction cost as a cost function [34,35,36]. However, it is necessary to recognize the impact of future uncertainties on optimization results and to consider the operation and maintenance (O&M) cost since both green and gray infrastructure have a long lifespan.

Consequently, the objectives of this study are to: (1) couple SWMM and NSGA-II to propose a simulation-optimization framework; (2) estimate the impact of various additional benefit levels of green infrastructure on optimization results of green-gray infrastructure; (3) optimize green, gray and green-gray infrastructures under future uncertainties.

A pilot site of Sponge City in Nanchang, China, was chosen as an example to achieve the above goals. Future climate change and urbanization scenarios were predicted by using General Circulation Models (GCMs), Change Factor Methodology (CFM), and district development plan, respectively.

## 2. Materials and Methods

### 2.1. Site Informations

**Figure 1:**
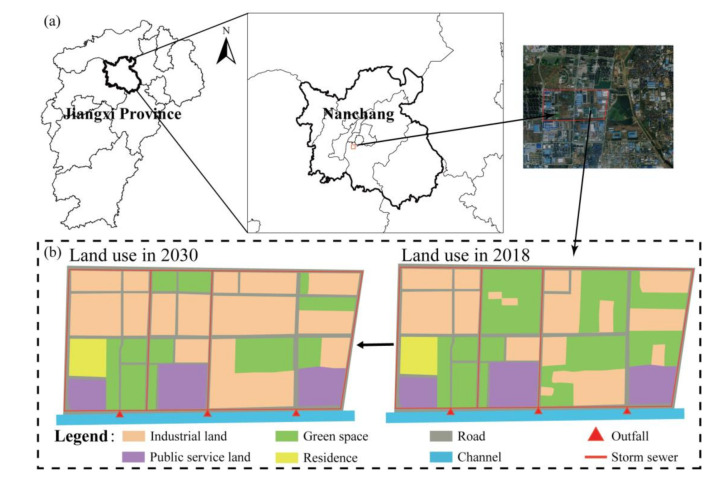
Information regarding the study area: (**a**) Location and scale of the study area; (**b**) Land use types currently (2018) and in the future (2030).

The study area is located in the south of Nanchang City, with an area of 152.2 ha. This site has a subtropical monsoon climate with an annual average rainfall of 1700 mm, and the rainy season is from May to July during each year, accounting for more than 50% of the total annual precipitation. In this site, polluted and untreated stormwater runoff has been discharged directly into poorly flowing man-made open channels through municipal separate storm sewer systems, and NPS poses a serious threat to the water bodies. Land use types in 2018 were extracted from GIS by referencing satellite images, and land use types in 2030 were simulated based on the Master Plan of Nanchang Central District (2011–2030) formulated by authorities. The main land use types in 2018 were industrial land and green space since the site is a developing economic development zone. In 2030, a number of green spaces will be turned into industrial land (Figure 1). Table 1 lists the specific information of land use types in 2018 and 2030.

### 2.2. SWMM Modeling

#### 2.2.1. Model Setup

**Figure 2:**
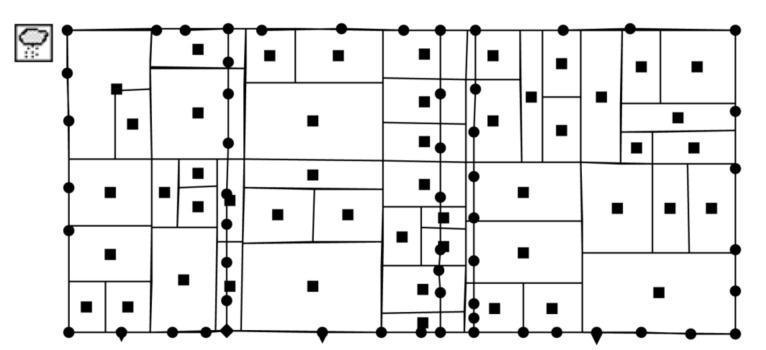
Generalization diagram of SWMM model.

To set up the SWMM model is the pre-processing step for optimization. The site was divided into 49 sub-catchments, 59 conduits, 54 nodes and 3 outfalls according to the extracted satellite images and the direction of the pipe networks (as shown in Figure 2). Horton infiltration was chosen for the hydrology module of SWMM, and the hydraulic module of SWMM was dynamic wave. TSS is one of the most representative pollutants in evaluating the water quality of runoff [37,38], and Assessment Standard for Sponge City Effect also requires to assess the water quality control effect by TSS removal rate [39], therefore, the pollutant in the water quality module of SWMM was set to be TSS, and the land uses in the water quality module were divided into roof, road/other impervious area and green space based on the surface features.

#### 2.2.2. Design Rainfall

The design rainfall event with the duration of 2 h and the return period of 2 years based on the Chicago approach was adopted in this study. The latest storm intensity equation in Nanchang is shown as below:

(1) $$i=\frac{9.5861\left(1+0.69\log P\right)}{{\left(t+1.4\right)}^{0.64}}$$

where *i* is rainfall intensity (mm/min); *P* is return period (year); *t* is rainfall duration (min).

#### 2.2.3. Goodness-of-Fit Test

SWMM has many parameters that are difficult to be determined directly, such as Max/Min infiltration rate, Manning’s coefficient for impervious/pervious area and conduits, depth of depression storage on impervious/pervious area, etc., in the hydrology module, buildup and washoff functions in the water quality module. Therefore, the parameters that are hard to be determined directly were first set referring to the SWMM Manual and past studies [25], and then, those were revised manually according to the past realistic rainfall events. Nash–Sutcliffe efficiency coefficient (NSE) is a commonly used method to test the goodness-of-fit between observed values and simulated results of hydrological models and was chosen to evaluate the accuracy of the model in this study. The NSE should be greater than 0.5, and the closer to 1 the NSE is, the more credible the model is.

(2) $$\mathrm{NSE}=1-\frac{\sum_{t=1}^{n}{\left(Q_{t}^{O}-Q_{t}^{S}\right)}^{2}}{\sum_{t=1}^{n}{\left(Q_{t}^{O}-{\bar{Q}}_{t}^{O}\right)}^{2}}$$

### 2.3. Setup Optimization Schemes

#### 2.3.1. Green Infrastructure and Gray Infrastructure

**Figure 3:**
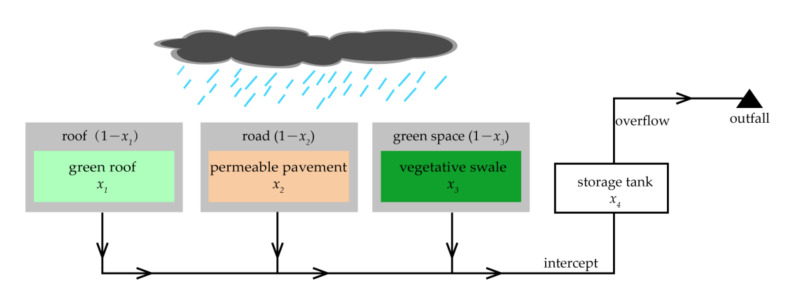
Designed scheme for green and gray infrastructure.

According to the land use types and surface condition in the study area, green roof, permeable pavement and vegetative swale were selected as green infrastructures to control the runoff from the source. Green roof, permeable pavement and vegetative swale were only placed on the roof, road and green space, respectively. Storage tanks were selected as the gray infrastructures and were set at the front of the three outfalls to intercept polluted runoff during the rainfall event at the terminal until the storage tank was full, and then the polluted runoff would discharge into the channel directly. The green-gray infrastructures scheme is shown in Figure 3.

#### 2.3.2. Decision Variables and Optimization Objectives

Changing the scale of infrastructures is the most significant factor to affect cost and performance compared to changing other parameters [18]. Thus, in order to make the optimization feasible and save optimization time, the decision variables for green infrastructure were construction area (*x*_1_, *x*_2_, *x*_3_) (see Figure 3) for each LID practice on a given land use type of each sub-catchment, and the other parameters were set as constant. The decision variables for gray infrastructure were construction area (*x*_4_) (see Figure 3) of storage tanks, and the height of storage tanks was kept as constant either.

We chose Life Cycle Cost (LCC) as an indicator to evaluate the economic benefits of green and gray infrastructure in this study. LCC includes construction cost and future O&M cost, and the future O&M cost is usually calculated by Discounted Cashflow Model (DCF), which can discount the expected future cost to the present cost and has been used in many previous studies [33,40,41]. Additionally, green infrastructure will produce additional benefits in its life cycle compared with gray infrastructure, such as ecological, aesthetic and environmental benefits [8], and this is why it has become the more recommended stormwater management at this stage. DCF was also used to calculate these future Value of Additional Benefits (VAB) of green infrastructure in this study. However, it is beyond the scope of this study to accurately assess the VAB. Therefore, this study used the α coefficient to represent the discounted values of the expected additional benefits in the future, and it can be considered as a positive cost to discount the negative LCC [36]. When the α coefficient is higher, there are more additional benefits that are taken into account.

How to obtain a higher NPS control rate in a lower cost as much as possible is a primary task in this study. Therefore, the LCC of the green and gray infrastructure and the TSS reduction rate were set to be minimizing and maximizing objectives, respectively.

The objective functions are shown as below:

(3) $$\begin{array}{ll} \min F_{1} & =\frac{C_{green}+V_{green}}{\alpha }\\ & =\frac{\sum_{i=1}^{n}\sum_{j=1}^{m}\sum_{k=1}^{o}A_{i}{}_{j}\cdot P_{j}{}_{k}\cdot N_{k}\left(1+\sum_{t=1}^{T_{green}}\frac{r}{{\left(1+v\right)}^{t}}\right)}{\alpha } \end{array}$$

(4) $$\begin{array}{ll} \min F_{2} & =C_{gray}+V_{gray}\\ & =\sum_{g=1}^{h}X_{g}\cdot H\cdot G\left(1+\sum_{t=1}^{T_{gray}}\frac{r}{{\left(1+v\right)}^{t}}\right) \end{array}$$

(5) $$\max F_{3}=1-\left(\frac{TSS}{TSS^{\prime }}\right)$$

where $C_{green}$ and $C_{gray}$ are the construction costs of green infrastructures and gray infrastructures, respectively; $V_{green}$ and $V_{gray}$ are the present discounted value of the O&M cost of green infrastructures and gray infrastructures, respectively; $A_{i}{}_{j}$ is the area of *j*th land use in *i*th sub-catchment; $P_{j}{}_{k}$ is the ratio of *k*th green infrastructure to *j*th land use; $N_{k}$ is the unit construction cost of *k*th green infrastructure; $X_{g}$ is the construction area of *g*th storage tank; *H* is the given height of storage tanks; *G* is the unit construction cost of storage tanks; *r* is the ratio of Annual O&M cost to the construction cost; *v* is the discount rate; $T_{green}$ and $T_{gray}$ are the life cycle of green and gray infrastructures; *n* is the number of subcatchments, *n* = 49; *m* is the types of land use, *m* = 3; *o* is the types of LID practices, *o* = 3; α is the coefficient representing the VAB of green infrastructure; TSS and $TSS^{\prime }$ extracted from SWMM by using Python are the TSS load discharged from the three outfalls before and after constructed green and gray infrastructures, respectively. The cost information is shown in Table 2.

The cost information of green and gray infrastructures was based on Technical Guide for Sponge City Construction and realistic projects in cities of the same economic level as Nanchang.

### 2.4. Future Scenario

Since both green and gray infrastructure have a long lifespan, it should not be neglected that urbanization and climate change in the future will have effects on its performance. Therefore, we designed four scenarios (C0U0, C30U0, C0U30, C30U30) in this study. The baseline scenario (C0U0) is land use in 2018, and the urbanization scenario (C0U30) is land use in 2030 according to the regional development plan. By comparing the differences between C0U0 and C0U30, we can conclude that green space will decrease from 35.38% (in 2018) to 19.67% (in 2030), while industrial land and road will increase from 32.18% (in 2018) to 45.64% (in 2018) and from 17.26% (in 2030) to 19.51% (in 2030), respectively (See Figure 1b). Therefore, the impervious ratio and land use type of the subcatchments in the SWMM model were changed correspondingly to simulate the urbanization scenario. Climate change scenarios (C0U0 and C30U0) were obtained by integrated Global Circulation Models (GCMs) and Change Factor Methodology (CFM), which has been validated and applied in the past studies [25,42]. MarkSim is a weather generator that emulates downscaling results from 17 GCMs for the four Representative Concentration Pathway (RCP) scenarios, including RCP 2.6, RCP 4.5, RCP 6.0, and RCP 8.5 [43]. It can create a random series of local daily precipitation in the future through given specific longitude and latitude coordinates. In this study, all of the GCMs were applied to obtain the ensemble average, and the RCP 6.0 scenario was selected because it is highly consistent with the current tendency and is the most likely to happen [44]. The process that uses CFM to estimate the local scaled future rainfall event is explained as follows [45]:

The first step is to use the GCMs weather generator to obtain daily precipitations in 2015 (baseline) and in 2030 (future);

Step 2 is to calculate the change factor (Equation (6));

Step 3 is to calculate the local scaled future scenario by applying change factors (Equation (7));

(6) $$CF=\frac{GCMs_{f}}{GCMs_{b}}$$

(7) $$f_{LS}=CF\cdot b_{LS}$$

where CF is change factor; $GCMs_{b}$ and $GCMs_{f}$ are the values of annual average precipitation from GCMs baseline and GCMs future climate scenario, respectively; $b_{LS}$ and $f_{LS}$ are the local scaled baseline and future values, respectively, and $b_{LS}$ is the design rainfall event by the storm intensity formula revised in 2015 based on the Chicago approach. The change factor was 1.16 in 2030.

### 2.5. Simulation-Optimization Framework

**Figure 4:**
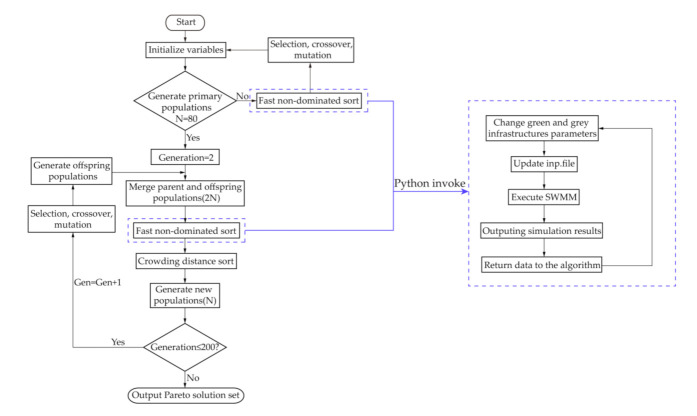
Simulation-optimization framework integrated by NSGA-Ⅱ and SWMM.

NSGA-II was used in this study because it is a stable, fast and accurate MOO algorithm [46], and it was widely used in LID practices layout optimization [29]. SWMM lacks in an optimization module, which makes it difficult to recognize the optimal solution from the many simulation results. In this simulation-optimization framework, we used Python to interfere the parameters and extract the simulation results from outputs, and the process was embedded in the workflow of NSGA-II. To make the solutions distributed uniformly and save computing time, the parameters of NSGA-II were set as follows: crossover probability = 0.95; mutation probability = 0.05; number of iterations = 300, and number of populations = 80. The simulation-optimization framework is shown in Figure 4.

## 3. Results and Discussion

### 3.1. Calibration and Validation

In this study, the calibration and validation for the model of hydrology module were implemented by comparing the simulated and observed runoff volume at the outfall during the rainfall event, while those of water quality module were implemented by comparing the simulated and observed TSS concentrations at the outfall during the rainfall events.

**Figure 5:**
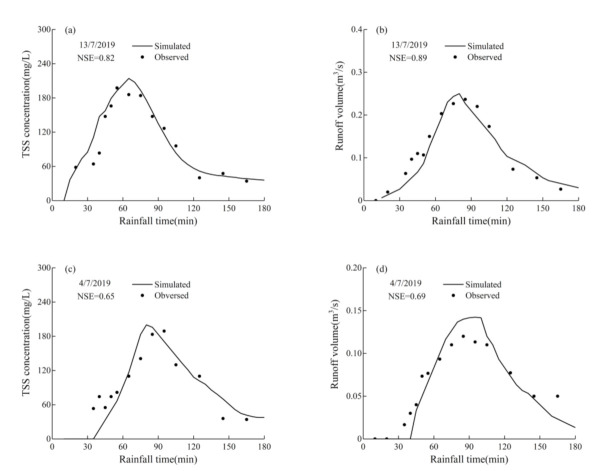
Comparison of simulated and observed values during rainfall events: (**a**) Calibration for TSS concentration; (**b**) Calibration for runoff volume; (**c**) Validation for TSS concentration; (**d**) Validation for runoff volume.

The rainfall event on 13 July 2019 was used to calibrate the model, and the rainfall event on 4 July 2019 was used to validate the results. Figure 5 shows the comparison of the simulated values and the observed values. The NSE values for calibration of the hydrology and water quality modules of the model were 0.89 and 0.82, respectively, while those of validation were 0.69 and 0.65, respectively. The NSE values of runoff volume for both calibration and validation are greater than those of TSS concentration, and the accuracy of the model for runoff volume simulation is greater than that for TSS concentration. In summary, the calibration and validation values for both hydrology module and water quality module are greater than 0.5, and it indicates that the accuracy of the model is acceptable, which can be applied to further following studies. The results of calibration for hydrology and water quality parameters of the model are shown in Table 3andTable 4.

### 3.2. Effect of VAB of Green Infrastructure on Optimizing

**Figure 6:**
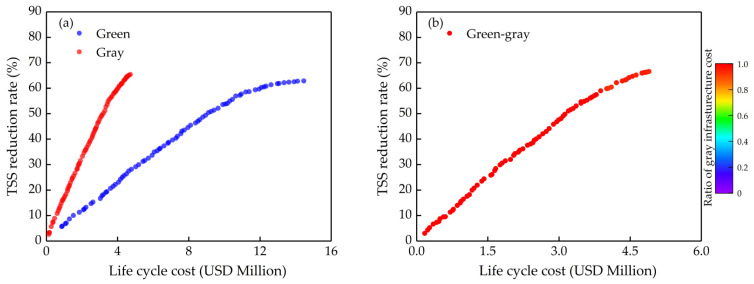
Results of Pareto solution set curves when the α coefficient is 1: (**a**) Separate optimizing (green infrastructure only and gray infrastructure only); (**b**) Synchronous optimizing (green-gray infrastructure).

**Figure 7:**
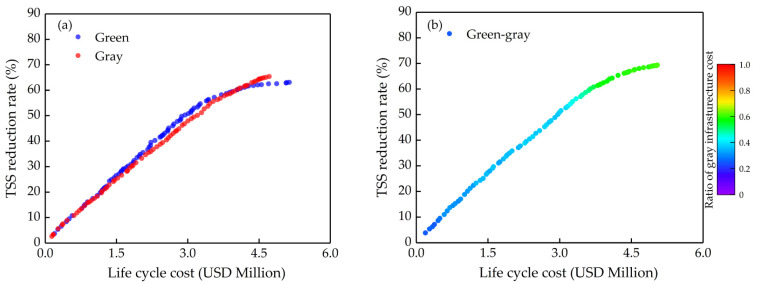
Optimization results when the α coefficient is 3: (**a**) Separate optimizing (green infrastructure only and gray infrastructure only); (**b**) Synchronous optimizing (green-gray infrastructure).

**Figure 8:**
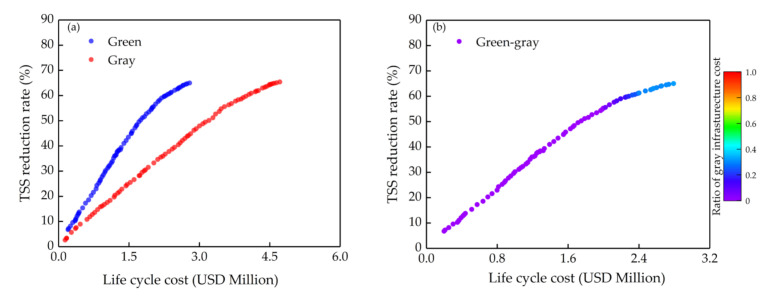
Pareto solution set curves when the α coefficient is 5: (**a**) Separate optimizing (green infrastructure only and gray infrastructure only); (**b**) Synchronous optimizing (green-gray infrastructure).

In this section, we mainly discussed the effects of different α coefficients on optimizing. The results are shown in Figure 6, Figure 7 and Figure 8. The green and the gray in the figures (Figure 6, Figure 7 and Figure 8a) are optimizing green infrastructure and gray infrastructure separately, while the green-gray is synchronously optimizing both green and gray infrastructure, and the color scale at the right of the graph is the ratio of gray infrastructure cost to total cost. Each point in Pareto solution sets obtained by multi-objective optimization is the optimal solution, which has the best benefit at a certain cost, and the solutions in the sets also contain a lot of information, such as the area of the green infrastructure in each sub-catchment and the volume of the gray infrastructure, which is helpful for decision makers to choose the most suitable solution intuitively based on water quality objectives and cost constraint.

The optimization results when α = 1 are shown in Figure 6. The cost-effectiveness curve of gray infrastructure is significantly different from green infrastructure (Figure 6a). The algorithm will not identify the additional benefits of green infrastructure when α = 1, and gray infrastructure is a more cost-effective stormwater management measure at this point. When synchronously optimizing both green and gray infrastructure, the algorithm would select the gray infrastructure almost exclusively in the range of the solution set interval. Therefore the gray infrastructure accounts for most of the cost weight of the green-gray infrastructure (Figure 6b). Due to the limited treatment capacity of gray infrastructure, its marginal benefit decreases gradually with the increasing cost, and the green infrastructure would be present slightly at TSS reduction rate above 60%. Although green infrastructure is a more promoted stormwater management measure, it is an inefficient measure for NPS control compared to gray infrastructure when its VAB were not considered. It means that gray infrastructure should be given priority as an NPS control measure when the investment is constrained.

When α = 3, the optimization results are shown in Figure 7. At this point, the green infrastructure shows a comparable performance to the gray infrastructure (Figure 7a). Since green and gray infrastructure treatment capacity is limited, their solution set curves tend to flatten gradually with the increasing cost, which is more pronounced for green infrastructure, and the finding is very similar to Leng et al. and Qiu et al. [25,31]. This means that the marginal return decreases gradually with increasing investment.

It can favor decision makers to choose a reasonable range of investment and thus to avoid inefficiencies. In optimizing process, the green and gray infrastructure showed a close probability of being selected by the algorithm. For example, the cost share of gray infrastructure is approximately 0.36 when the TSS reduction rate is 50% (Figure 7b). However, the cost share of gray infrastructure will gradually increase with the increasing cost. This may be due to the weaker performance attenuation effect of gray infrastructure caused by increasing cost compared to green infrastructure so that the gray infrastructure accounts for an increasing proportion when the cost increases. In addition, the synchronous optimizing green-gray infrastructure can slow down the tendency of the curve to flatten compared to the single infrastructure, which makes the solution set curve be more linear. In other words, if green and gray infrastructure have similar cost-effectiveness, green-gray infrastructure will show the co-benefit, delaying the tendency of their marginal benefit to decline. This result is similar to Zhou et al. [36]. However, it is worth noting that the co-benefit is not obvious when the cost is not too high. For example, the TSS reduction rate is 50.6%, 48.3% and 50.8% for green, gray and green-gray infrastructure when the cost is USD 3 million, respectively, while the TSS reduction rate is 56.7%, 55.5% and 58.6% for green, gray and green-gray infrastructure when the cost is USD3.5 million, respectively (Figure 7). The co-benefit increases the possibility that watersheds reach the maximum NPS control rate and will gradually appear with the increasing cost.

The optimization results when α = 5 are shown in Figure 8. At this point, there are enough VAB to be considered, and obviously, green infrastructure is a more cost-effective NPS control measure than gray infrastructure (Figure 8a). Gray infrastructure would not be selected by the algorithm for green-gray infrastructure when the TSS reduction rate is below 51% (Figure 8b). However, since green infrastructure has higher attenuation performance caused by increasing cost, consistent with the discussion above, a better solution at this point as the green infrastructure is that the gray infrastructure would still be considered when the TSS reduction rate is above 51%. In summary, when α = 5, decision makers could choose green infrastructure as a principal stormwater management measure. Only when a higher water quality objective needs to be reached should a supplement of a certain scale of gray infrastructure on the basis of constructed green infrastructure be considered.

### 3.3. Optimizing Green and Gray Infrastructure under Future Uncertainties

In this section, we chose α = 5 as the VAB of the green infrastructure for the following analysis of green and gray infrastructure under future uncertainties, for green infrastructure has a comparable cost-effectiveness to gray infrastructure at this additional benefits level as discussed in the last section.

#### 3.3.1. Impact of Water Quality under Future Uncertainties

The TSS load discharged from the outfalls under different scenarios are shown in Table 5. Compared to C0U0, C30U0, C0U30 and C30U30, the TSS load increases by 2.71%, 5.26% and 9.92%, respectively. The change of impervious ratio and the increased pollutant load on the surface under urbanization scenario have a greater impact on TSS load than the higher wash-off effect caused by the increased rainfall intensity under climate change scenario. In addition, urbanization and climate change have a synergistic rather than a linear effect on TSS load, the finding is similar to previous studies [1]. This means that the change in land use and climate in the future poses a serious threat to the water ecosystem by producing more NPS.

#### 3.3.2. Optimization Results under Future Uncertainties

**Figure 9:**
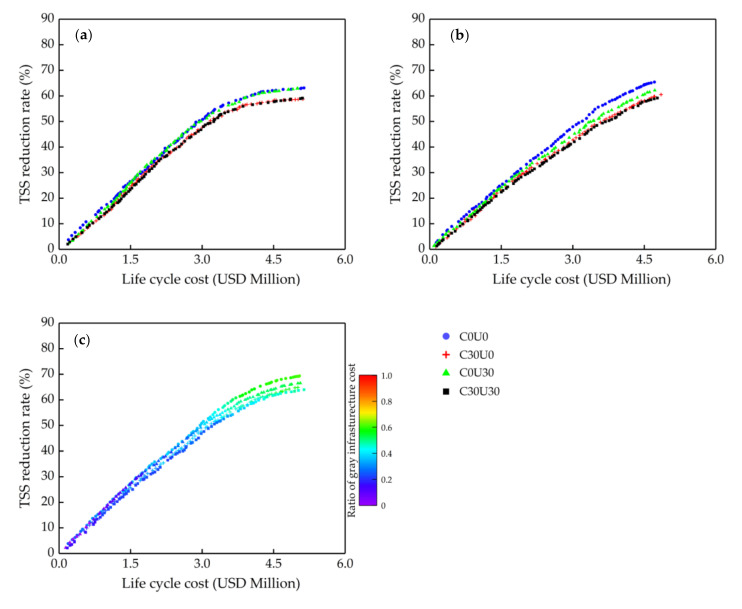
Pareto solution set curves under four future scenarios: (**a**) Optimizing green infrastructure only; (**b**) Optimizing gray infrastructure only; (**c**) Optimizing green-gray infrastructure.

Figure 9 shows the optimization results under the four scenarios. By observing the Pareto solution sets, we found that both green and gray infrastructure are more sensitive to climate change than urbanization (see Figure 9a,b).

For example, at USD 4 million, the TSS reduction rates for green and gray infrastructure are approximately 59.6% and 59.8% under C0U0, respectively, but 56.7% and 53.8% under C30U0, respectively. This is due to the green and gray infrastructure which is mainly relied on to reduce the runoff volume and thus to control the TSS load. Under C30U0, the green and gray infrastructure were filled up earlier by the increased runoff volume generated from the higher intensity rainfall, and those have a greater impact on storage tanks.

According to the optimization results under C0U30, although there is more runoff generated from increasing ratio of impervious surface under C0U30, the concentration of TSS in polluted runoff will also be higher with increasing TSS load on the surface caused by the change of land use, so the green and gray infrastructure can remove more TSS load at a certain runoff volume reduction rate compared to those under C30U0. In addition, it is worth noting that the Pareto solution curve of green infrastructure under C0U30 is almost unchanged. Besides the reasons discussed above, another reason is the phenomenon that green infrastructure was placed targetedly on critical areas with high TSS load resulting from urbanization. In other words, in optimizing process, the algorithm reallocated cost and placed green infrastructure at the source, that is, new critical areas with high TSS load generated from the change of land use type. However, gray infrastructure cannot be placed on the new areas caused by urbanization with high TSS load. The finding elucidated that gray infrastructure is more sensitive under urbanization compared to green infrastructure. In practice, storage tanks are a huge project and difficult to continue expanded with the urbanization process, while LIDs are a small project placed at source, and decision makers can continually adjust their planning schemes to suit the process of urbanization.

Figure 9c depicts the variation in ratio of gray infrastructure cost in green-gray synchronous optimization results. For example, when the cost is USD 3 million, the ratio of gray infrastructure cost is 0.39, 0.35, 0.33 and 0.27 under C0U0, C30U0, C0U30 and C30U30, respectively. Compared to C0U0, gray infrastructure is less selected in other future scenarios. When the cost is below USD 2.93 million, the curve C0U30 and the curve C0U0 almost overlap, and green infrastructure is more selected compared to other future scenarios, while the curve C0U30 cannot maintain the same increasing trend as the curve C0U0 with the increasing cost. This can be explained by the differences in the sensitivity of green and gray infrastructure under future uncertainties which can affect the synchronous optimization results of green and gray infrastructure. In other words, the same construction planning of green-gray infrastructure under C0U0 is not the optimal solution under other future scenarios. The finding indicates that to adapt the urbanization and climate change by adjusting the combination strategy of green and gray infrastructure can obtain a higher resilience in future uncertainties.

As a whole, the green-gray infrastructure shows comparable resilience in future uncertainties with the green infrastructure. For example, when the cost is USD 4 million, the TSS reduction rates for green and green-gray infrastructure are approximately 59.6% and 63.2% under C0U0, respectively, but 55.1% and 58.4% under C30U30. However, as discussed in the last section, the marginal benefit of green-gray infrastructure decreases more slowly with increasing cost than green infrastructure only. Therefore, green-gray infrastructure should be considered mainly if green infrastructure has a similar resilience to green-gray infrastructure under future uncertainties.

#### 3.3.3. Maintaining Water Quality under Future Impact

**Figure 10:**
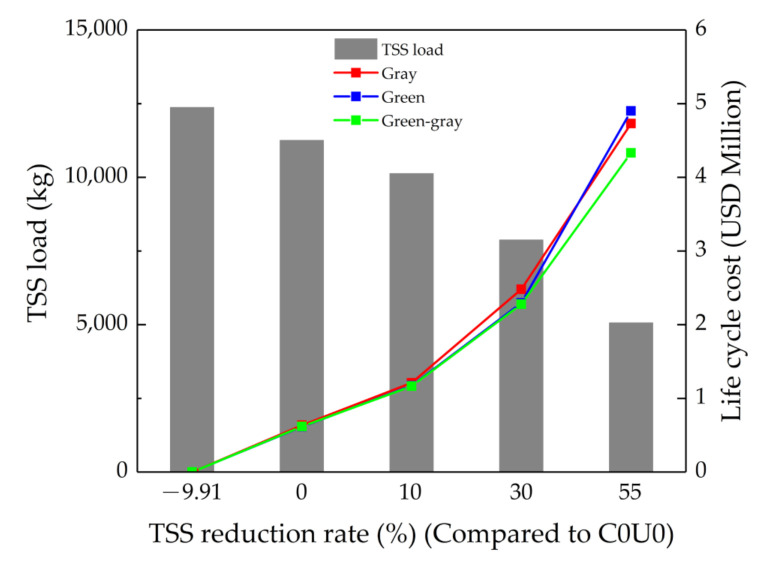
Costs of maintaining the water quality levels to C0U0 under C30U30.

Figure 10 shows the costs of maintaining the water quality levels to C0U0 under C30U30 (based on the optimization results). In the *X*-axis, −9.91% is the TSS load under C30U30 (increase of 9.91% compared to C0U0), and 0, 10, 30 and 55 are the same level as C0U0 and reductions by 10%, 30% and 55% compared to those of C0U0, respectively. The first values of cost are USD 0, meaning there are no control measures to be implemented under C30U30. The second values of cost mean that it will cost USD 0.635, USD 0.617 and USD 0.614 million to maintain the same water quality of C0U0 under C30U30 for gray, green and green-gray infrastructure, respectively, and at this level of water quality objective, the co-benefit of the green-gray infrastructure are less pronounced. The third values of cost are USD 1.211, USD 1.172 and USD 1.165 million for gray, green and green-gray infrastructure, respectively. The fourth values of cost are USD 2.482, USD 2.295 and USD 2.278 million for gray, green and green-gray infrastructure, respectively. The fifth values of cost are USD 4.730, USD 4.902 and USD 4.332 million for gray, green, and green-gray infrastructure, respectively, and at this level of water quality objective, gray infrastructure is a more cost-effective measure than green infrastructure. In addition, the co-benefit of green-gray infrastructure is relatively obvious. Figure 10 indicates that as the TSS load decreases, the cost increases markedly. However, the increase of cost with TSS load decreasing is various for green, gray, and green-gray infrastructure, and the cost-effectiveness of green-gray infrastructure is higher than that of green or gray infrastructure only. Therefore, when the low control objectives need to be reached, green infrastructure should be considered as the main measure, while gray infrastructure should be the main measure when the control objectives are relatively high.

## 4. Conclusions

In this study, a simulation-optimization framework was developed by integrated NSGA-Ⅱ and SWMM to optimize green and gray infrastructure for NPS control under climate change and urbanization scenarios. Considering the VAB of green infrastructure, different values of α coefficients were applied to assess the impact on optimization results. The following conclusions were obtained:

1. Gray infrastructure is a more cost-effective measure for NPS control, unless there are sufficient VAB of green infrastructure to be considered. When a high NPS control rate is reached, the marginal benefit of green infrastructure decreases faster than that of gray infrastructure, so when the water quality objective is relatively high, it is a less economical measure to adopt only green infrastructure for NPS control.
2. When there are enough VAB (i.e., α = 3) for the green infrastructure, green-gray infrastructure will show co-benefits compared to green or gray infrastructure only, and the co-benefit will be more obvious with the increasing cost.
3. Green and green-gray infrastructure have a greater resilience under future uncertainties, especially under urbanization scenario, while gray infrastructure is more sensitive under climate change scenario.

The results of this study can serve as a reference for the cost-effectiveness issues involved in watershed NPS control. However, the VAB of green infrastructure was not accurately determined in this study, and it is a relatively simplified approach to represent the VAB green infrastructure by α coefficients. In addition, the gray infrastructure optimization scheme is also a relative simplification, and only the volume of storage tanks is considered in optimizing. Changing the location of the storage tanks may have an impact on its performance. Therefore, in the further following exploration, a method to accurately evaluate the VAB of green infrastructure needs to be established, and both location and volume of storage tanks should be optimized.

## Figures and Tables

**Table 1:** Land use information in 2018/2030.

Land Use	2018	2030
Industrial land	32.18%	45.64%
Green space	35.38%	19.67%
Road	17.26%	19.51%
Public service land	11.98%	11.98%
Residence	3.20%	3.20%

**Table 2:** The cost information of green and gray infrastructure in the study area.

Infrastructure Types	Construction Cost (USD)	Annual O&M Cost (Ratio of Construction Cost)	Life Cycle (Year)	Discount Rate
green roof	54.39/m^2^	0.03	25	0.05
permeable pavement	35.74/m^2^	0.02	25	0.05
vegetative swale	31.08/m^2^	0.02	25	0.05
storage tank	388.5/m^3^	0.05	20	0.05

**Table 3:** Hydrology parameters of the model.

Parameters	Physical Meaning	Values
MaxRate (mm/h)	Maximum infiltration rate	77.5
MinRate (mm/h)	Minimum infiltration rate	4.9
Decay (1/h)	Infiltration decay rate	2
Dry time (days)	Time for a fully saturated soil to completely dry	7
N-imperv	Manning’s N for impervious areas	0.012
N-perv	Manning’s N for pervious areas	0.12
Dstore-imperv (mm)	Depression storage on impervious areas	0.15
Dstore-perv (mm)	Depression storage on pervious area	1.15

**Table 4:** Buildup and washoff parameters of the model.

	Exponential Buildup Function		Exponential Washoff Function	
Land Use Types	Max. Buildup (kg/ha)	Rate Constant (day)	Washoff Coefficient	Washoff Exponent
Roof	167.5	0.3	0.006	1.8
Green space	162.2	0.6	0.004	1.4
Road/other impervious area	191.4	0.3	0.006	1.8

**Table 5:** TSS load under different future scenarios.

Scenarios	C0U0	C30U0	C0U30	C30U30
TSS load (kg)	11259	11546	11851	12376
Increase rate	0	2.71%	5.26%	9.92%

## Data Availability

Not applicable.

## References

[1] Shrestha S., Bhatta B., Shrestha M., Shrestha P.K. (2018). Integrated assessment of the climate and landuse change impact on hydrology and water quality in the Songkhram River Basin, Thailand. Sci. Total Environ..

[2] Zhou Q., Leng G., Su J., Ren Y. (2019). Comparison of urbanization and climate change impacts on urban flood volumes: Importance of urban planning and drainage adaptation. Sci. Total Environ..

[3] Hu D., Zhang C., Ma B., Liu Z., Yang X., Yang L. (2020). The characteristics of rainfall runoff pollution and its driving factors in Northwest semiarid region of China—A case study of Xi’an. Sci. Total Environ..

[4] Le T.T.A., Lan-Anh N.T., Daskali V., Verbist B., Vu K.C., Anh T.N., Nguyen Q.H., Nguyen V.G., Willems P. (2021). Urban flood hazard analysis in present and future climate after statistical downscaling: A case study in Ha Tinh city, Vietnam. Urban Water J..

[5] Yazdanfar Z., Sharma A. (2015). Urban drainage system planning and design—Challenges with climate change and urbanization: A review. Water Sci. Technol..

[6] Fan M., Shibata H. (2015). Simulation of watershed hydrology and stream water quality under land use and climate change scenarios in Teshio River watershed, northern Japan. Ecol. Indic..

[7] Wang M., Sun Y., Sweetapple C. (2017). Optimization of storage tank locations in an urban stormwater drainage system using a two-stage approach. J. Environ. Manag..

[8] Eckart K., McPhee Z., Bolisetti T. (2017). Performance and implementation of low impact development—A review. Sci. Total Environ..

[9] Zhang P., Cai Y., Wang J. (2018). A simulation-based real-time control system for reducing urban runoff pollution through a stormwater storage tank. J. Clean. Prod..

[10] Rong G.W., Hu L.Y., Wang X., Jiang H.L., Gan D.N., Li S.S. (2021). Simulation and evaluation of low-impact development practices in university construction: A case study of Anhui University of Science and Technology. J. Clean. Prod..

[11] Sambito M., Severino A., Freni G., Neduzha L. (2021). A Systematic Review of the Hydrological, Environmental and Durability Performance of Permeable Pavement Systems. Sustainability.

[12] Jia H.F., Wang X.W., Ti C.P., Zhai Y.Y., Field R., Tafuri A.N., Cai H.H., Yu S.L. (2015). Field monitoring of a LID-BMP treatment train system in China. Environ. Monit. Assess..

[13] Maniquiz-Redillas M.C., Kim L.H. (2016). Evaluation of the capability of low-impact development practices for the removal of heavy metal from urban stormwater runoff. Environ. Technol..

[14] Wilson C.E., Hunt W.F., Winston R.J., Smith P. (2015). Comparison of Runoff Quality and Quantity from a Commercial Low-Impact and Conventional Development in Raleigh, North Carolina. J. Environ. Eng..

[15] Yang W., Wang Z., Hua P., Zhang J., Krebs P. (2021). Impact of green infrastructure on the mitigation of road-deposited sediment induced stormwater pollution. Sci. Total Environ..

[16] Luan B., Yin R., Xu P., Wang X., Yang X., Zhang L., Tang X. (2019). Evaluating Green Stormwater Infrastructure strategies efficiencies in a rapidly urbanizing catchment using SWMM-based TOPSIS. J. Clean. Prod..

[17] Sun Y., Deng L., Pan S.Y., Chiang P.C., Shah K.J.J.W.-E.N. (2020). Integration of green and gray infrastructures for sponge city: Water and energy nexus. Water Energy Nexus.

[18] Ho H.C., Lin S.W., Lee H.Y., Huang C.C. (2019). Evaluation of a Multi-Objective Genetic Algorithm for Low Impact Development in an Overcrowded City. Water.

[19] Zhu Z.H., Chen X.H. (2017). Evaluating the Effects of Low Impact Development Practices on Urban Flooding under Different Rainfall Intensities. Water.

[20] Hua P., Yang W., Qi X., Jiang S., Xie J., Gu X., Li H., Zhang J., Krebs P. (2020). Evaluating the effect of urban flooding reduction strategies in response to design rainfall and low impact development. J. Clean. Prod..

[21] Feng M., Jung K., Li F., Li H., Kim J.-C. (2020). Evaluation of the Main Function of Low Impact Development Based on Rainfall Events. Water.

[22] Dong X., Guo H., Zeng S. (2017). Enhancing future resilience in urban drainage system: Green versus grey infrastructure. Water Res..

[23] Alves A., Gersonius B., Kapelan Z., Vojinovic Z., Sanchez A. (2019). Assessing the Co-Benefits of green-blue-grey infrastructure for sustainable urban flood risk management. J. Environ. Manag..

[24] Li J., Wang Y., Ni Z., Chen S., Xia B. (2020). An integrated strategy to improve the microclimate regulation of green-blue-grey infrastructures in specific urban forms. J. Clean. Prod..

[25] Leng L., Jia H., Chen A.S., Zhu D.Z., Xu T., Yu S. (2021). Multi-objective optimization for green-grey infrastructures in response to external uncertainties. Sci. Total Environ..

[26] Wang J., Liu J.H., Wang H., Mei C. (2020). Approaches to Multi-Objective Optimization and Assessment of Green Infrastructure and Their Multi-Functional Effectiveness: A Review. Water.

[27] Saadatpour M., Delkhosh F., Afshar A., Solis S.S. (2020). Developing a simulation-optimization approach to allocate low impact development practices for managing hydrological alterations in urban watershed. Sustain. Cities Soc..

[28] Raei E., Alizadeh M.R., Nikoo M.R., Adamowski J. (2019). Multi-objective decision-making for green infrastructure planning (LID-BMPs) in urban storm water management under uncertainty. J. Hydrol..

[29] Men H., Lu H., Jiang W.J., Xu D. (2020). Mathematical Optimization Method of Low-Impact Development Layout in the Sponge City. Math. Probl. Eng..

[30] Eckart K., McPhee Z., Bolisetti T. (2018). Multiobjective optimization of low impact development stormwater controls. J. Hydrol..

[31] Qiu S., Yin H.W., Deng J.L., Li M.H. (2020). Cost-Effectiveness Analysis of Green-Gray Stormwater Control Measures for Non-Point Source Pollution. Int. J. Environ. Res. Public Health.

[32] Saldarriaga J., Salcedo C., Solarte L., Pulgarin L., Rivera M.L., Camacho M., Iglesias-Rey P.L., Martinez-Solano F.J., Cunha M. (2020). Reducing Flood Risk in Changing Environments: Optimal Location and Sizing of Stormwater Tanks Considering Climate Change. Water.

[33] Bakhshipour A.E., Dittmer U., Haghighi A., Nowak W. (2019). Hybrid green-blue-gray decentralized urban drainage systems design, a simulation-optimization framework. J. Environ. Manag..

[34] Li C.L., Liu M., Hu Y.M., Han R.Q., Shi T., Qu X.Q., Wu Y.L. (2018). Evaluating the Hydrologic Performance of Low Impact Development Scenarios in a Micro Urban Catchment. Int. J. Environ. Res. Public Health.

[35] Li F., Yan X.F., Duan H.F. (2019). Sustainable Design of Urban Stormwater Drainage Systems by Implementing Detention Tank and LID Measures for Flooding Risk Control and Water Quality Management. Water Resour. Manag..

[36] Zhou Q., Lai Z., Blohm A. (2019). Optimising the combination strategies for pipe and infiltration-based low impact development measures using a multiobjective evolution approach. J. Flood Risk Manag..

[37] Gong Y.W., Liang X.Y., Li X.N., Li J.Q., Fang X., Song R.N. (2016). Influence of Rainfall Characteristics on Total Suspended Solids in Urban Runoff: A Case Study in Beijing, China. Water.

[38] Zhang M.L., Chen H., Wang J.Z., Pan G. (2010). Rainwater utilization and storm pollution control based on urban runoff characterization. J. Environ. Sci..

[39] MHURD Assessment Standard for Sponge City Effect, the Ministry of Housing and Urban-Rural Development (MHURD) of People Republic of China (PRC). http://www.mohurd.gov.cn/wjfb/201904/t20190409_240118.html.

[40] Xu T., Engel B.A., Shi X., Leng L., Jia H., Yu S.L., Liu Y. (2018). Marginal-cost-based greedy strategy (MCGS): Fast and reliable optimization of low impact development (LID) layout. Sci. Total Environ..

[41] Zhou Q., Mikkelsen P.S., Halsnaes K., Arnbjerg-Nielsen K. (2012). Framework for economic pluvial flood risk assessment considering climate change effects and adaptation benefits. J. Hydrol..

[42] Liu Y., Theller L.O., Pijanowski B.C., Engel B.A. (2016). Optimal selection and placement of green infrastructure to reduce impacts of land use change and climate change on hydrology and water quality: An application to the Trail Creek Watershed, Indiana. Sci. Total Environ..

[43] Jones P.G., Thornton P.K. (2013). Generating downscaled weather data from a suite of climate models for agricultural modelling applications. Agric. Syst..

[44] Liu Y., Engel B.A., Collingsworth P.D., Pijanowski B.C. (2017). Optimal implementation of green infrastructure practices to minimize influences of land use change and climate change on hydrology and water quality: Case study in Spy Run Creek watershed, Indiana. Sci. Total Environ..

[45] Anandhi A., Frei A., Pierson D.C., Schneiderman E.M., Zion M.S., Lounsbury D., Matonse A.H. (2011). Examination of change factor methodologies for climate change impact assessment. Water Resour. Res..

[46] Deb K., Pratap A., Agarwal S., Meyarivan T. (2002). A fast and elitist multiobjective genetic algorithm: NSGA-II. IEEE Trans. Evol. Comput..

